# Retraction: Investigation of the biological activity, mechanical properties and wound healing application of a novel scaffold based on lignin–agarose hydrogel and silk fibroin embedded zinc chromite nanoparticles

**DOI:** 10.1039/d6ra90014c

**Published:** 2026-02-11

**Authors:** Reza Eivazzadeh-Keihan, Hooman Aghamirza Moghim Aliabadi, Fateme Radinekiyan, Mohammad Sobhani, Farzane khalili, Ali Maleki, Hamid Madanchi, Mohammad Mahdavi, Ahmed Esmail Shalan

**Affiliations:** a Catalysts and Organic Synthesis Research Laboratory, Department of Chemistry, Iran University of Science and Technology Tehran 16846-13114 Iran maleki@iust.ac.ir +98-21-73021584 +98-21-73228313; b Faculty of Chemistry, K. N. Toosi University of Technology Tehran Iran; c Protein Chemistry Laboratory, Department of Medical Biotechnology, Biotechnology Research Center, Pasteur Institute of Iran Tehran Iran; d Department of Biotechnology, School of Medicine, Semnan University of Medical Sciences Semnan Iran hamidmadanchi@yahoo.com; e Drug Design and Bioinformatics Unit, Department of Medical Biotechnology, Biotechnology Research Center, Pasteur Institute of Iran Tehran Iran; f Endocrinology and Metabolism Research Center, Endocrinology and Metabolism Clinical Sciences Institute, Tehran University of Medical Sciences Tehran Iran; g BCMaterials, Basque Center for Materials, Applications and Nanostructures, Martina Casiano, UPV/EHU Science Park Barrio Sarriena s/n Leioa 48940 Spain; h Central Metallurgical Research and Development Institute (CMRDI) P. O. Box 87, Helwan Cairo 11421 Egypt a.shalan133@gmail.com

## Abstract

Retraction of ‘Investigation of the biological activity, mechanical properties and wound healing application of a novel scaffold based on lignin–agarose hydrogel and silk fibroin embedded zinc chromite nanoparticles’ by Reza Eivazzadeh-Keihan *et al.*, *RSC Adv.*, 2021, **11**, 17914–17923, https://doi.org/10.1039/D1RA01300A.

The Royal Society of Chemistry hereby wholly retracts this *RSC Advances* article due to concerns with the reliability of the data.

There are concerns with the MTT plate image in Fig. 4B, the wound healing images in Fig. 5B and the hemolysis plate image in Fig. 6B, with these images being duplicated in other articles by the same authors.^1–3^

The authors’ response and data have been reviewed by an independent expert who has deemed them unsatisfactory.

Given the significance of these concerns, the Editor has lost confidence that the findings presented in this paper are reliable.

This retraction supersedes the information provided in the Expression of Concern related to this article.

Ahmed Esmail Shalan stated their role was limited to revising, editing and helping in the publishing process.

Farzane Khalili stated their role was limited to the chemistry-related aspects of the study, and had no role in the biological experiments or analyses.

Fateme Radinekiyan stated their role was limited exclusively to the chemistry section of the paper and had no involvement in the biological part of this study.

Hamid Madanchi states that his contribution to this work was limited to providing the laboratory bench, consumable materials, cell lines, and microbial strains. He does not agree with the retraction. He states that the MTT and hemolysis images associated with this article (Fig. 4B and 6B) were originally published in this *RSC Advances* paper and were later reused in other articles published subsequently, which have since been retracted. He further notes that all original raw images of the mouse and wound experiments, along with complete supporting documentation, were provided to the journal.

The authors were informed about the retraction of the article. Ahmed Esmail Shalan, Farzane Khalili and Fateme Radinekiyan have agreed with the retraction. Hamid Madanchi, Ali Maleki and Reza Eivazzadeh-Keihan have not agreed with the decision, the other authors have not indicated whether they agree with the decision to retract.

Signed: Ahmed Esmail Shalan, Farzane Khalili and Fateme Radinekiyan

Date: 30th January 2026

Retraction endorsed by Laura Fisher, Executive Editor, *RSC Advances*
